# Supplementary material for: FTO Intronic SNP Strongly Influences Human Neck Adipocyte Browning Determined by Tissue and PPARγ Specific Regulation: A Transcriptome Analysis
Source: Cells. 2020 Apr 16;9(4):987. doi: 10.3390/cells9040987 (PMC7227023; doi:10.3390/cells9040987)
Supplement: Supplementary file 1 [file cells-09-00987-s001.zip › Supplementary Figures Cells.docx]

**Supplementary information**


FTO intronic SNP strongly influences human neck adipocyte browning determined by tissue and PPARγ specific regulation: a transcriptome analysis


Beáta B. Tóth, Rini Arianti, Abhirup Shaw, Attila Vámos, Zoltán Veréb, Szilárd Póliska, Ferenc Győry, Zsolt Bacso, László Fésüs*, Endre Kristóf*

**Supplementary Figure 1. Functional features of the primary SC and DN adipocytes differentiated for two weeks to white or brown. (A) Basal, cAMP stimulated and oligomycin inhibited oxygen consumption (OC) levels** (as compared to basal OCR of SC white adipocytes) n=4. **(B)** **Basal and cAMP-stimulated extracellular acidification levels** (as compared to basal ECAR of SC white adipocytes) n=4. **(C)** **The proportion of creatine kinase futile cycle related OC at basal and cAMP-stimulated** n=4. SC: Subcutaneous, DN: Deep-neck, W: white differentiation protocol, B: brown differentiation protocol. Statistics: In comparison of two groups two-tailed paired Student’s t-test was used. *p<0.05. In multi-factor comparision we used two-way ANOVA and post hoc Tukey’s test. *p<0.05. **p<0.01. n=4


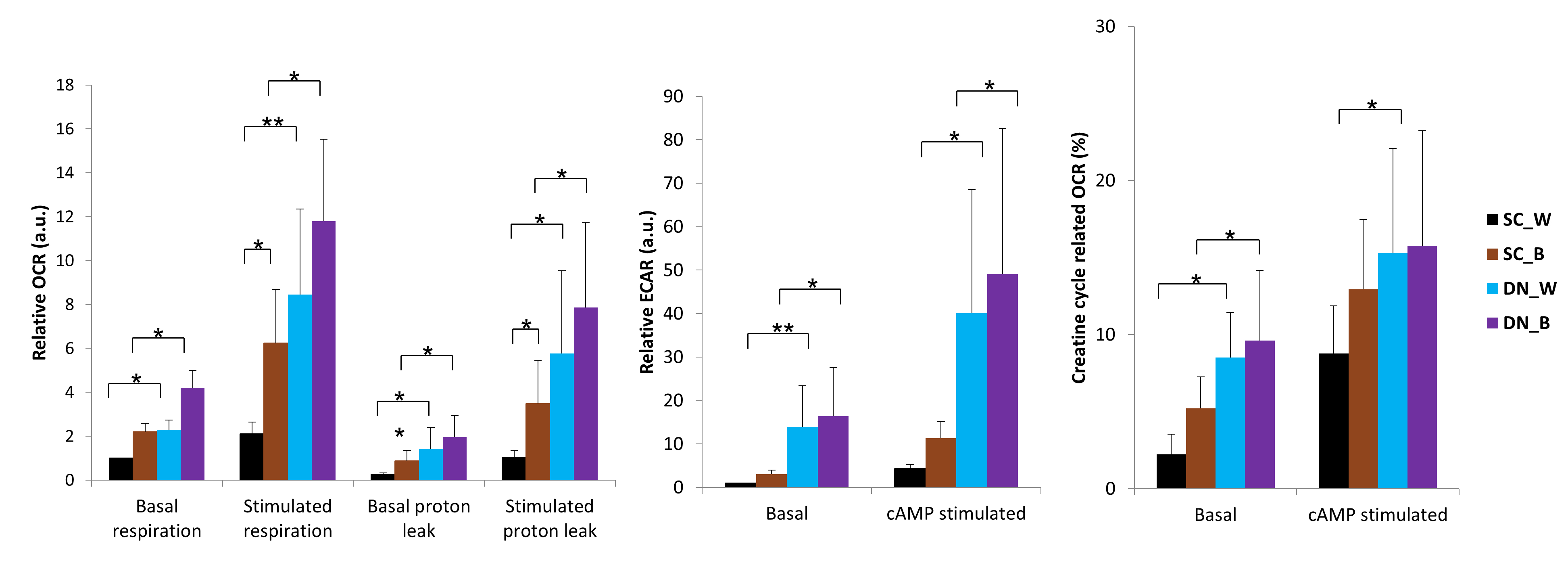


**C**

**B**

**A**

**Supplementary Figure 1.**


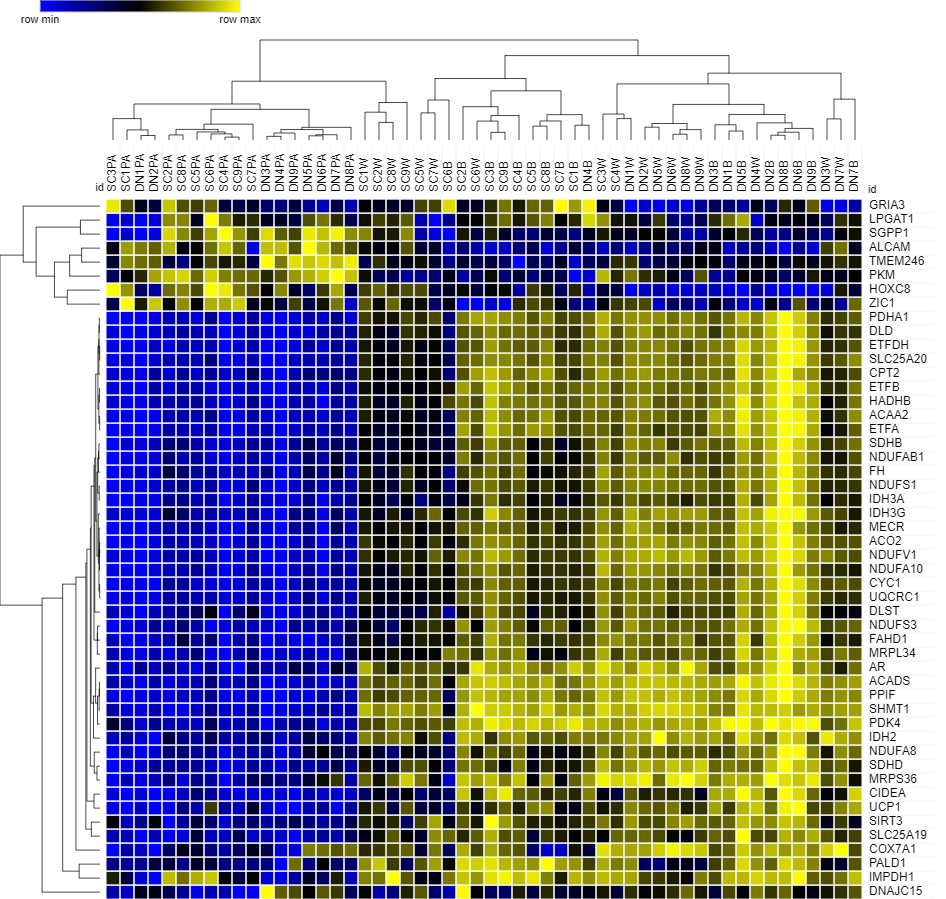

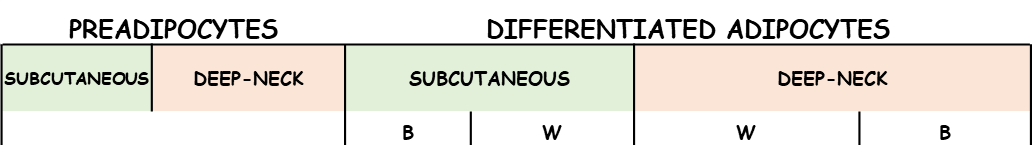


-**2.7 2.7**


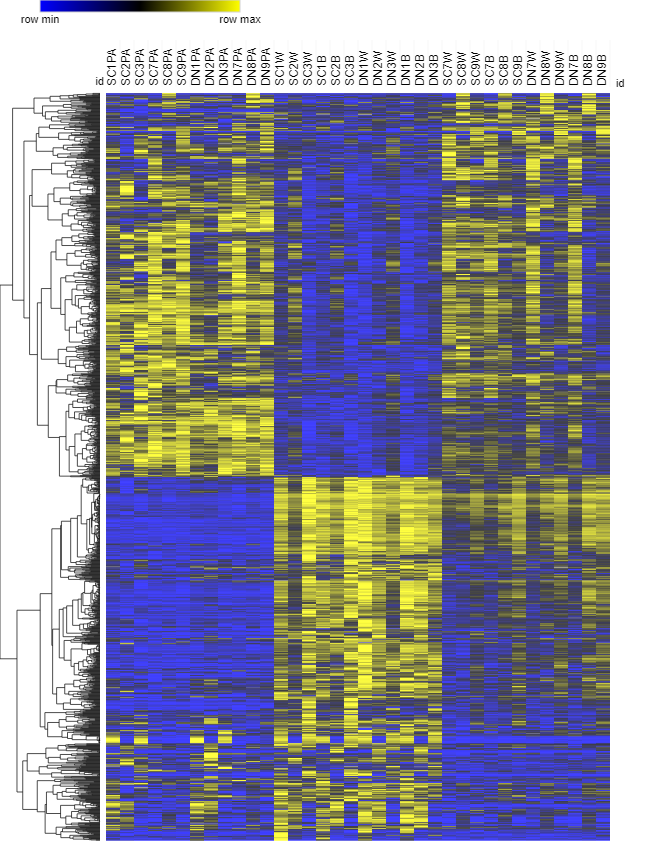

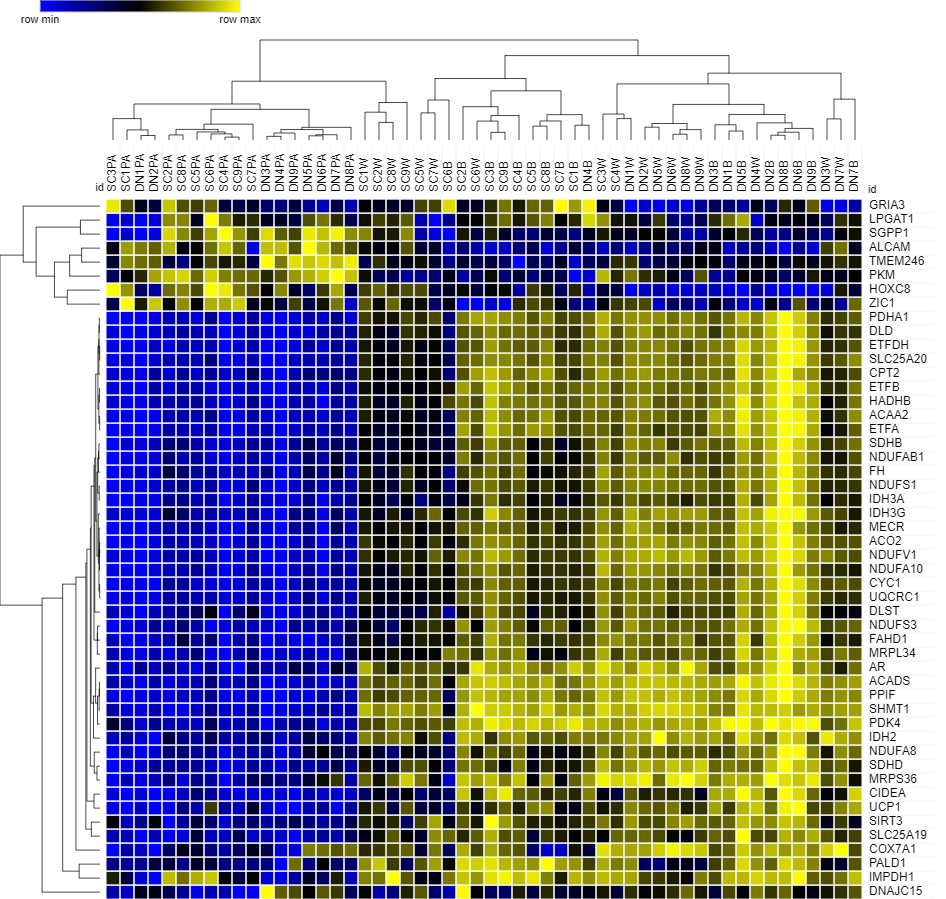


**Z-score by donor**

**Expression of ProFAT brown markers**

**Supplementary Figure 2. Expression profile of ProFAT marker genes.** Heat map shows the expression profile of browning (44) and white (6) characteristic marker genes from ProFAT database in SC and DN adipose progenitors and differentiated samples.

**Supplementary Figure 2.**

**Supplementary Figure 3. Gene-expression of key browning marker genes based on RNAseq data**. SC: Subcutaneous DN: Deep-neck. W: white differentiation protocol; B: brown differentiation protocol; Statistics: Deseq R data. *p<0.05. n=9 donors. 4 samples/donor.


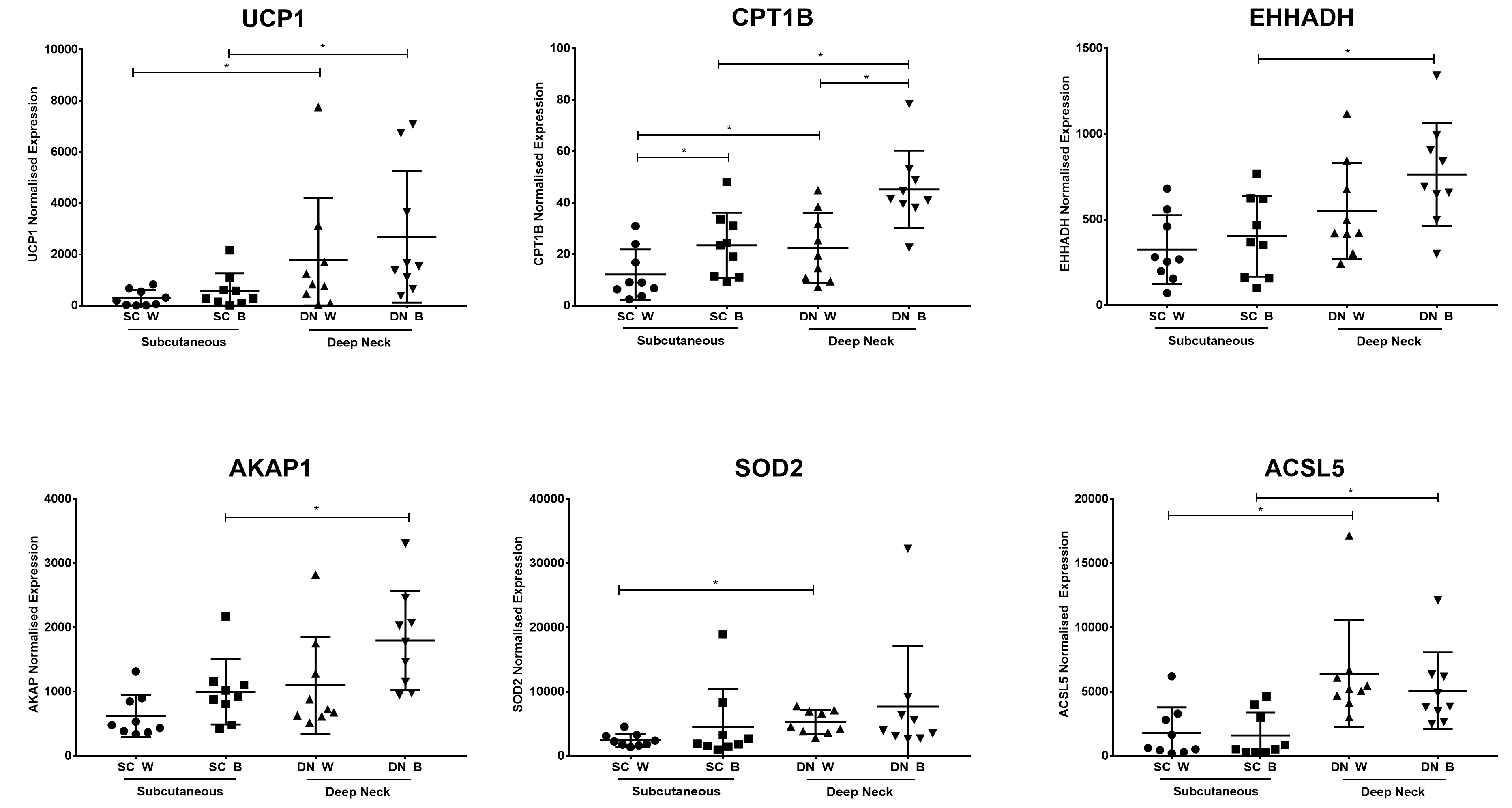

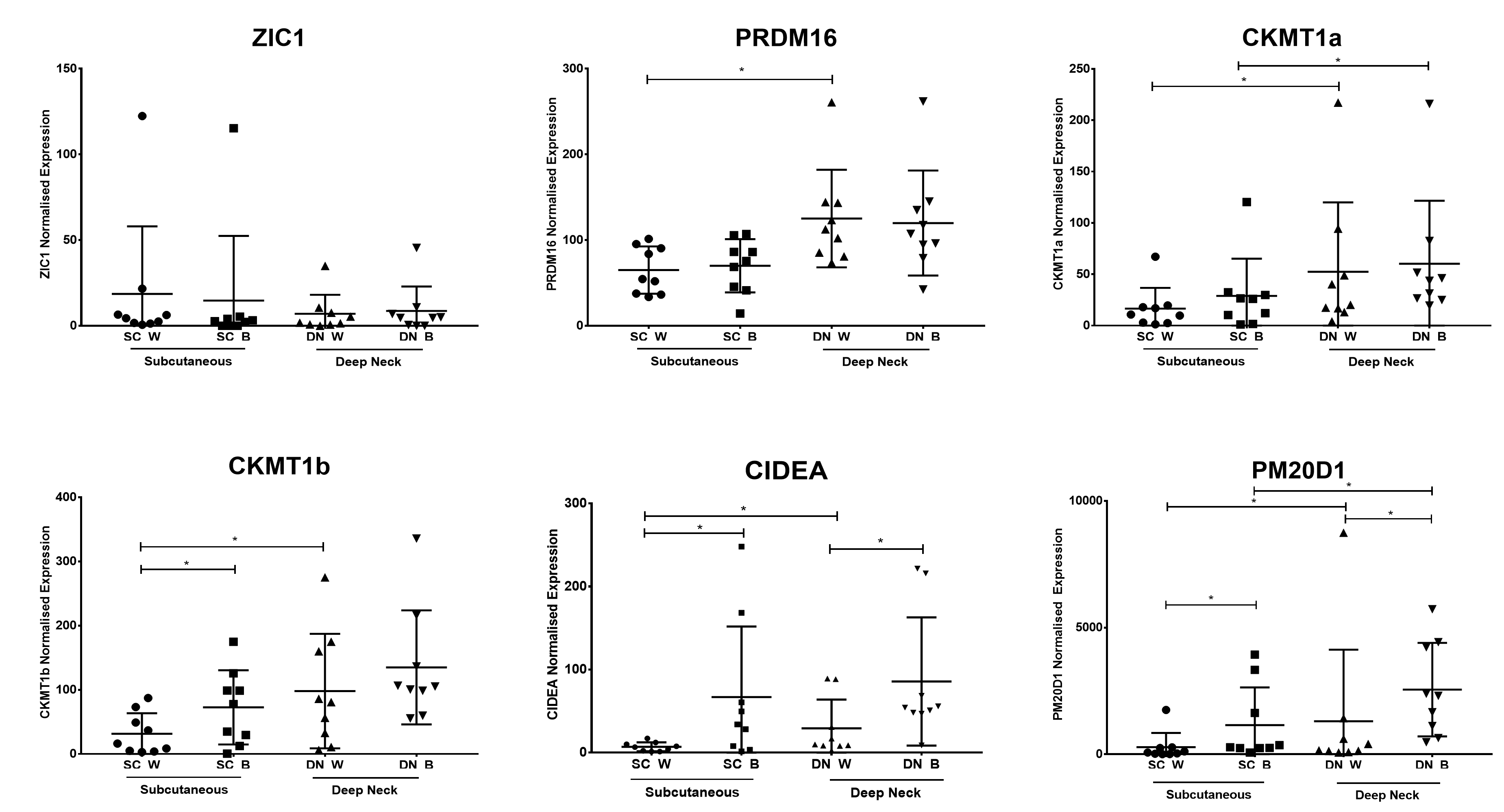


***ZIC1***

***PRDM16***

***CKMT1A***

***UCP1***

***CPT1B***

***EHHADH***

***AKAP1***

***SOD2***

***ACSL5***

***CKMT1B***

***CIDEA***

***PM20D1***

**Supplementary Figure 3.**


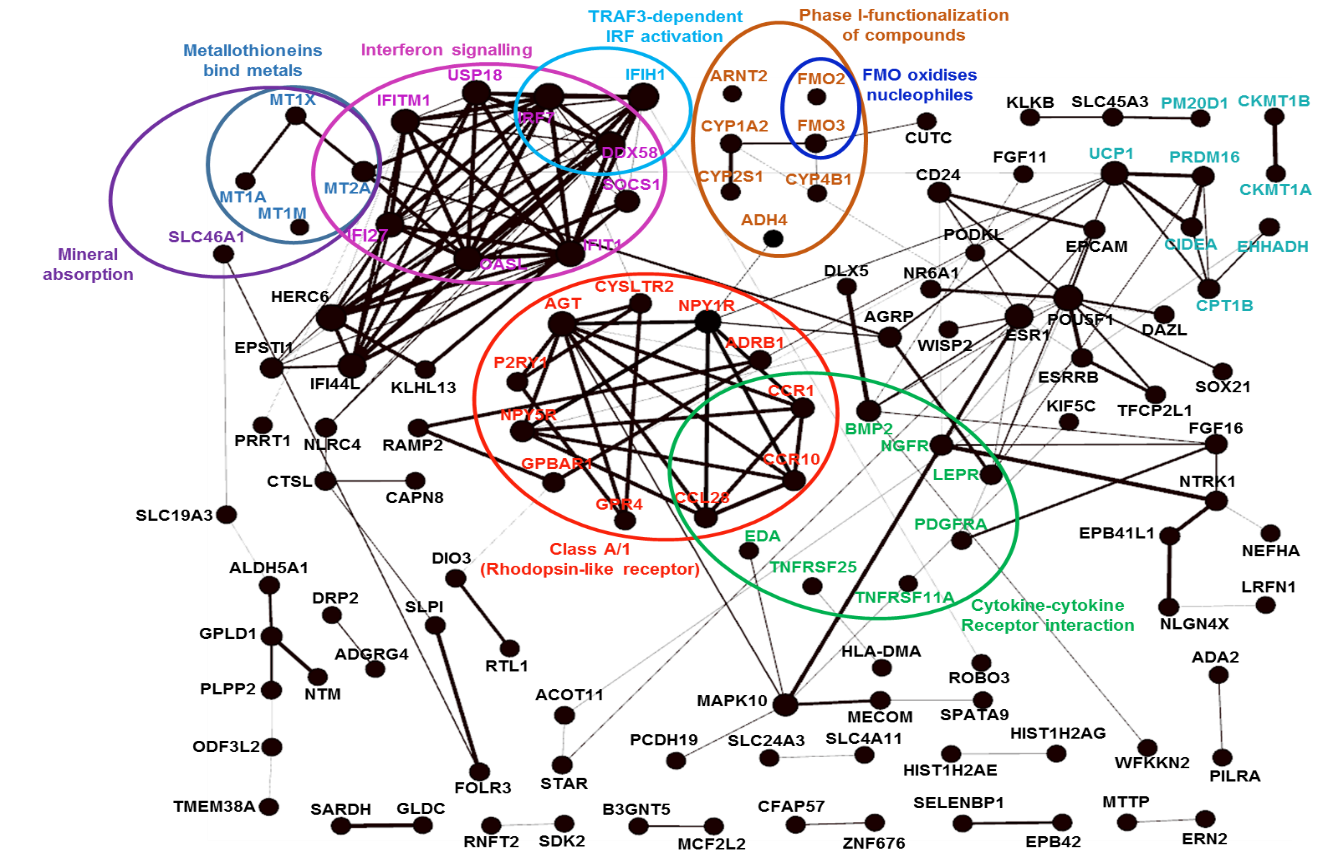

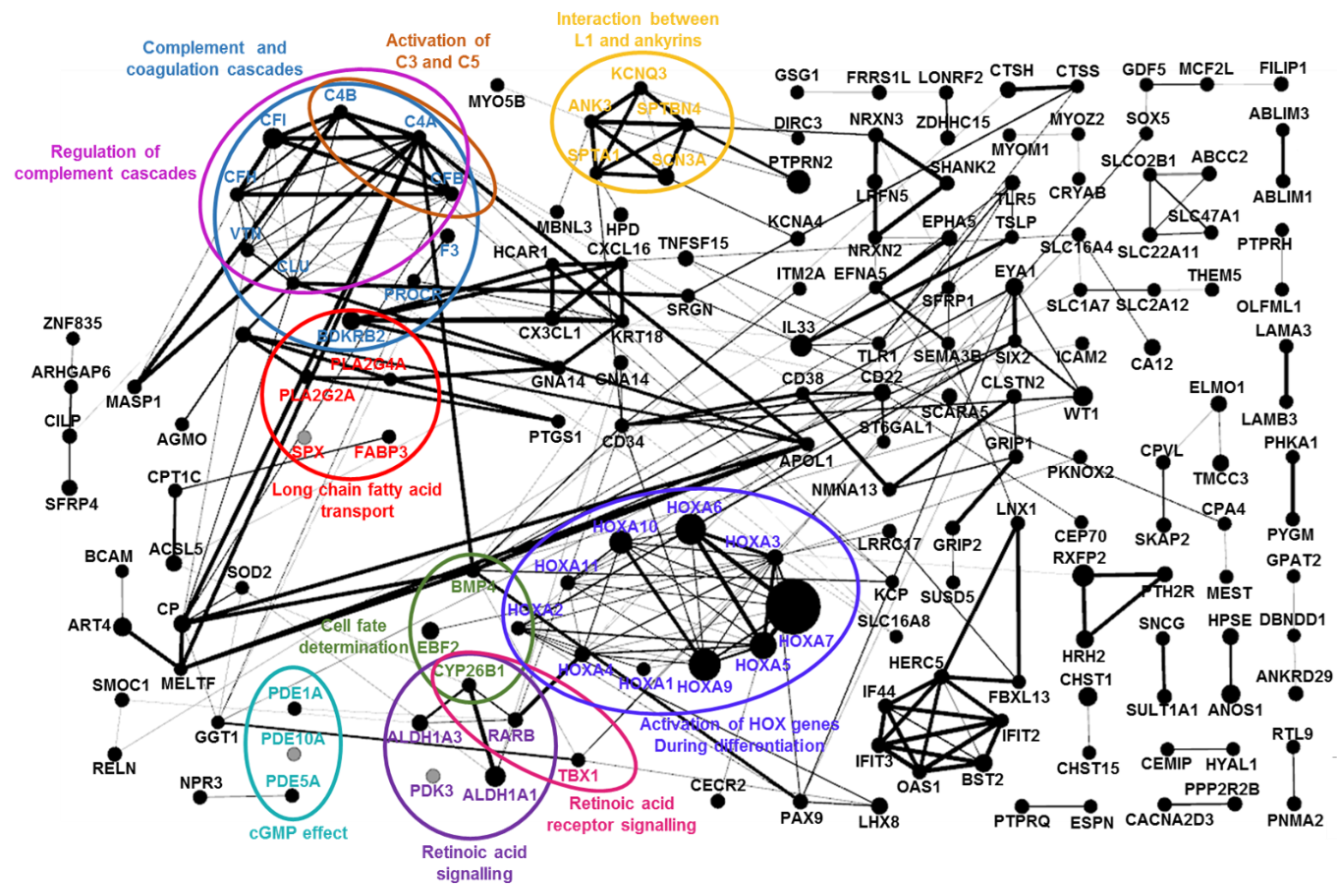


**A**

**B**

**Supplementary Figure 4.**


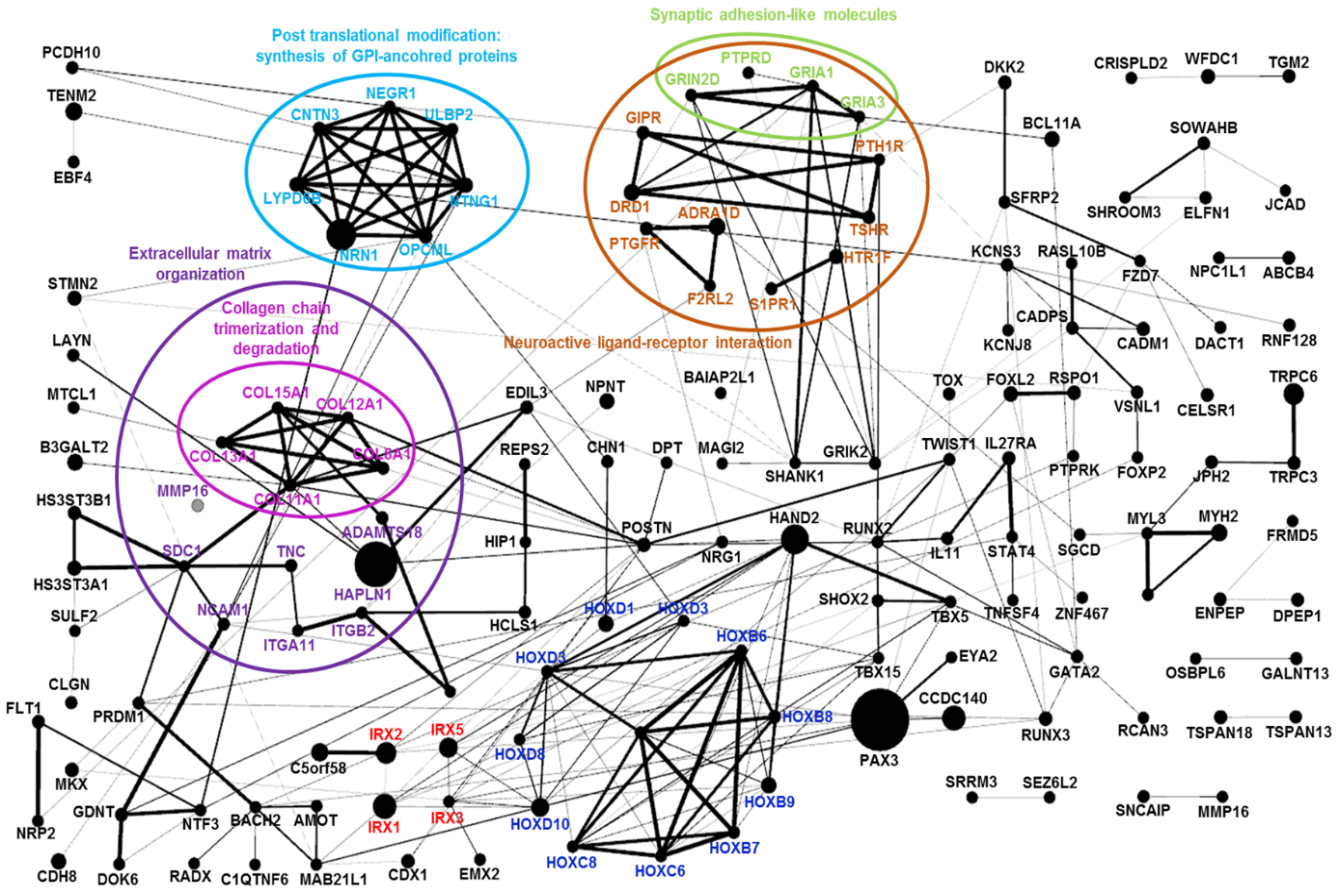

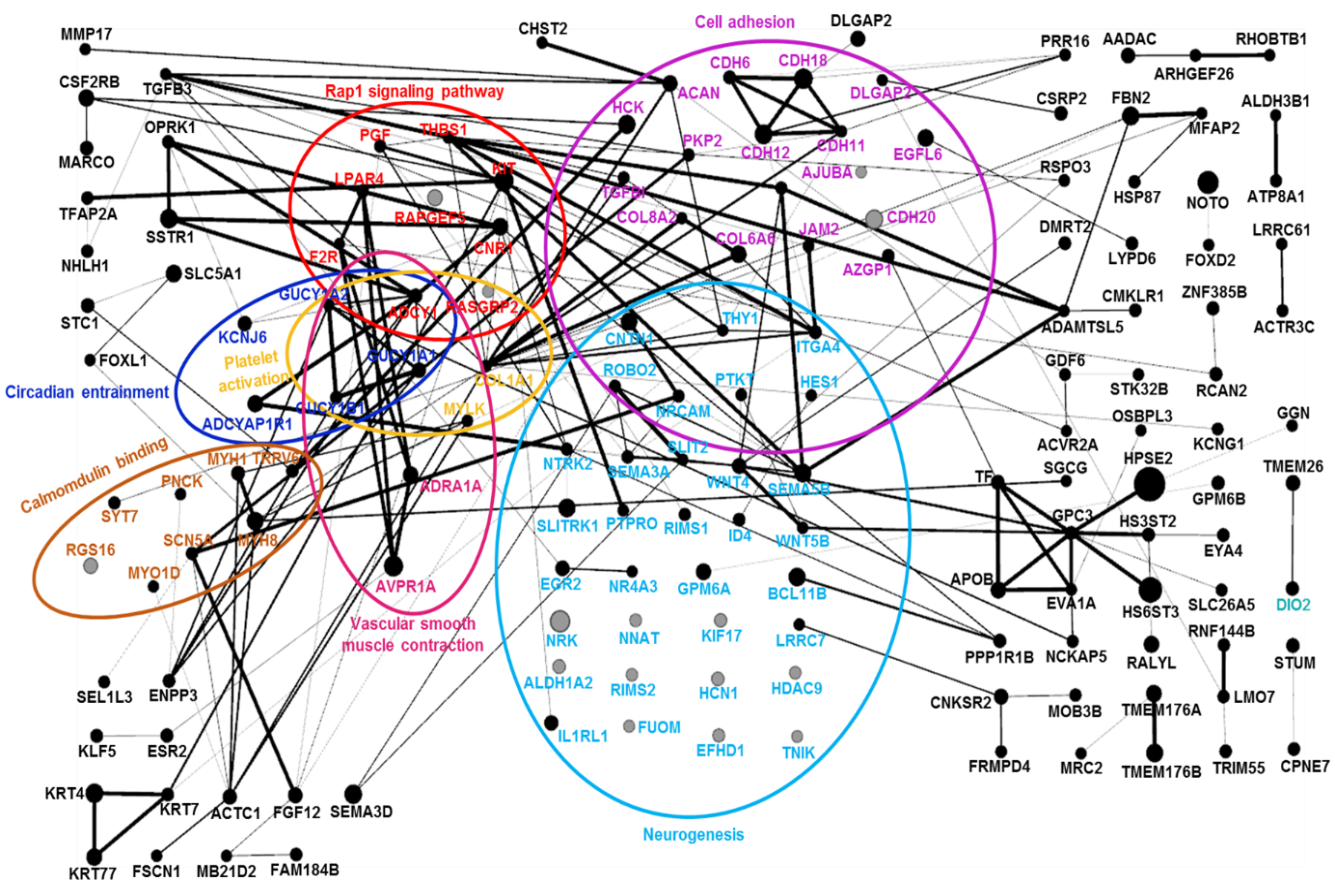


**D**

**C**

**Supplementary Figure 4. Interactome maps of group 1-4 genes** **(A) Interactome map of group 1 genes. (B) Interactome map of group 2 genes (C) Interactome map of group 3 genes (D) Interactome map of group 4 genes.** The interaction network was determined by STRING ([https://string-db.org](https://string-db.org/)) and constructed by using Gephi 0.9.2 ([https://gephi.org](https://gephi.org/)). Size of the nodes reflect the fold change of DN/SC (**A and B**) and SC/DN (**C and D**). Edges represent protein-protein interactions and the size of the edges indicate the confidence of interaction or the strenght of data support. The clustering was performed based on reactome (reactome.org) and KEGG Mapper ([www.kegg.jp](http://www.kegg.jp/)).

**Supplementary Figure 5. FTO obesity-risk allele presence based analyses of gene-expression profiles. (A) Expression profile of ProFAT marker genes**. Heatmap shows the expression profile of browning (44) and white (6) characteristic marker genes from ProFAT database in SC and DN adipose progenitors and differentiated samples; samples was hierarchically clustered based on pearson correlation. n=9 **(B) Expression profile of adipocyte specific BATLAS marker genes.** Heatmap shows the expression profile of browning (98) and white (21) characteristic marker genes from BATLAS database in SC and DN adipose progenitors and differentiated samples, samples was hierarchically clustered based on pearson correlation. To indentify donor differences z-score was calculated by considering all donors. n=9. **(C) Expression profile of the 81 genes encoding mitochondrial proteins**, which expressed differently according FTO obesity-risk allele presence. **(D) Expression profiles of mitochondrial proteins encoding genes based on Human MitoCarta 2.0 database.** Heat map shows the expression profiles of 1038 genes encoding mitochondrial proteins based on the presence of the *FTO* obesity-risk allele C/C in SC and DN adipose progenitors and differentiated samples. z-score was calculated by all samples to identify donor differences. n = 6 donors. SC Subcutaneous; DN: Deep-neck; W white differentiation protocol; B brown differentiation protocol. Numbers 1-9 represents donors, * outlier from the cluster.

**A**

**D**

**C**

**B**


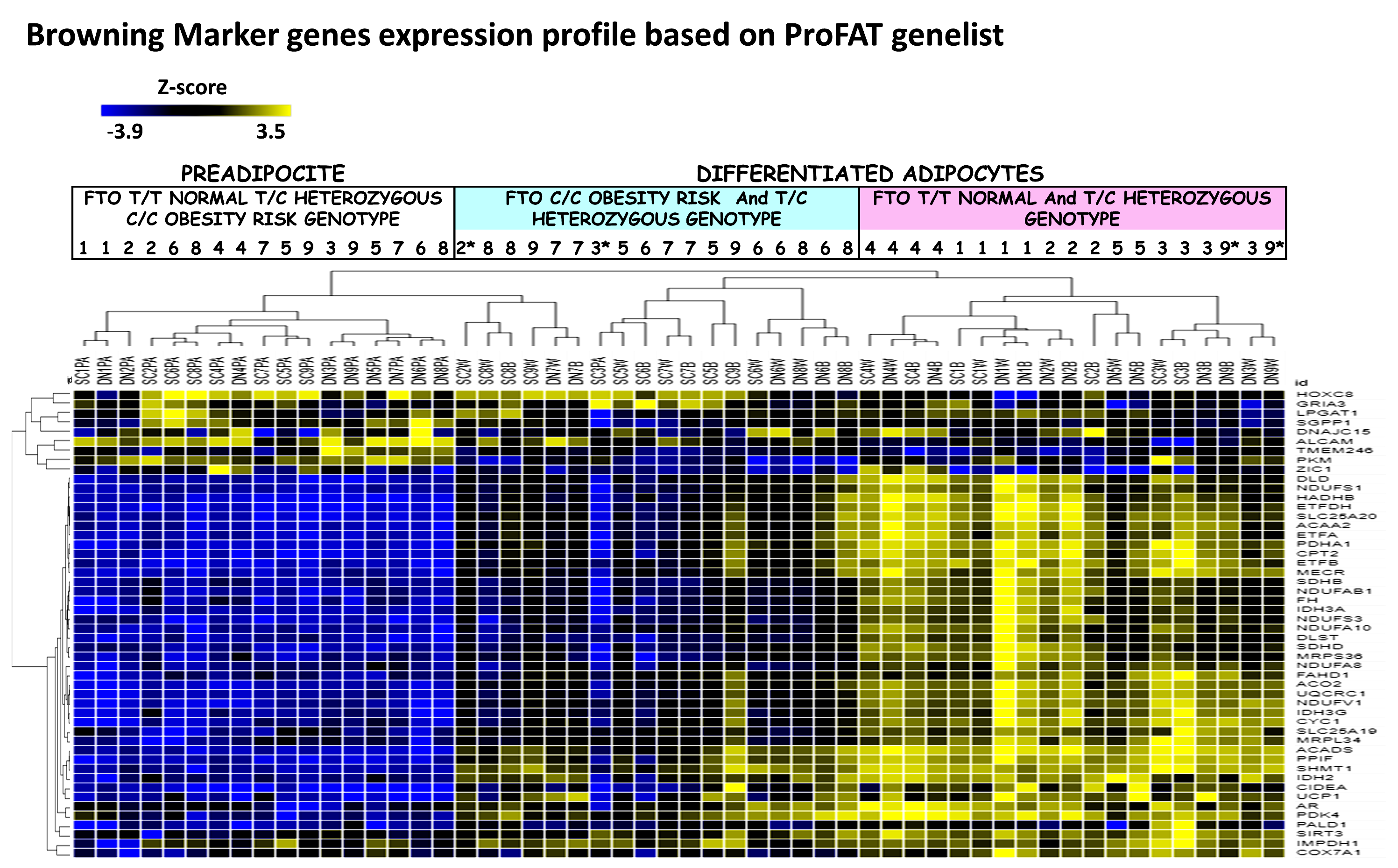

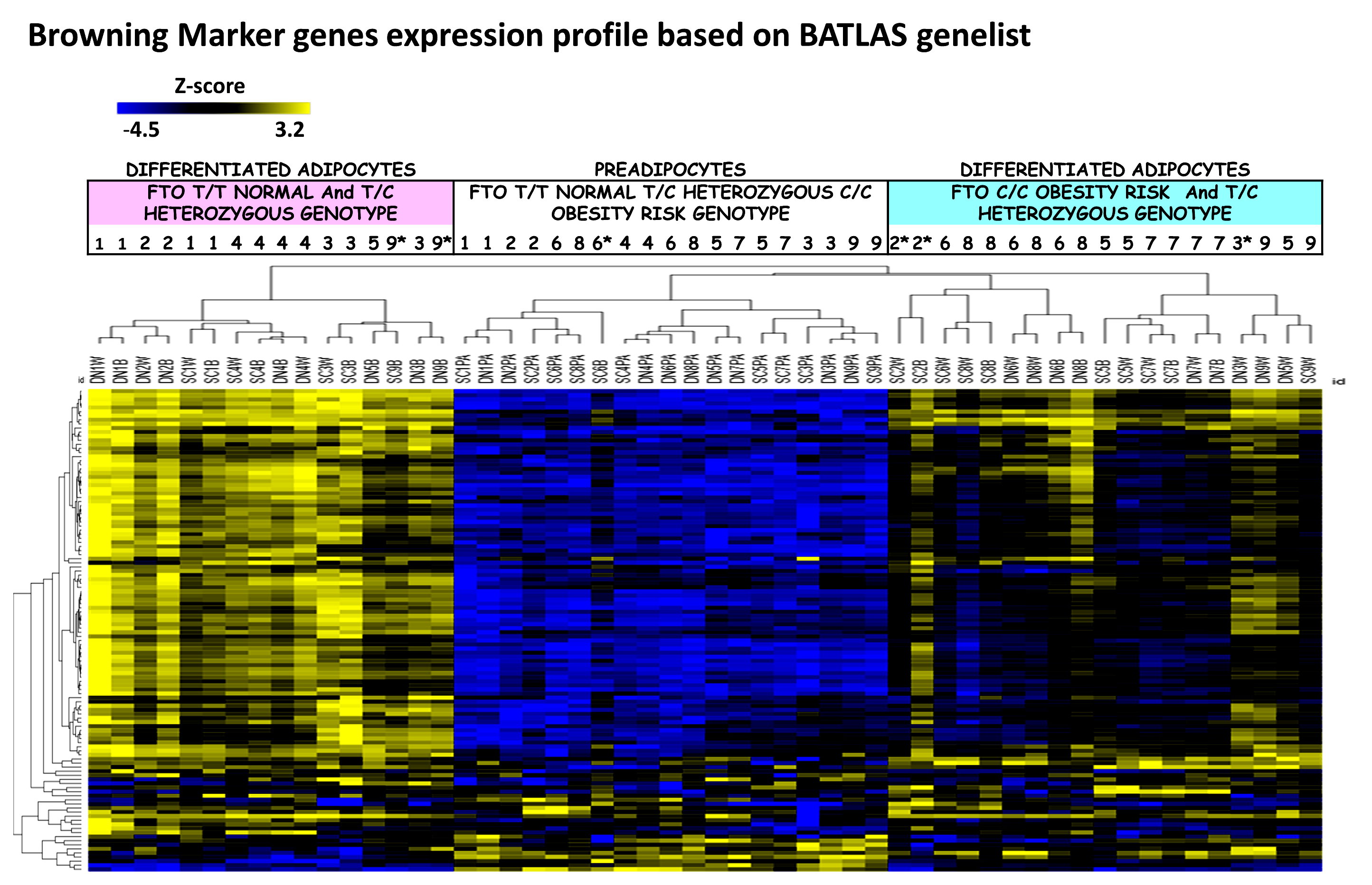

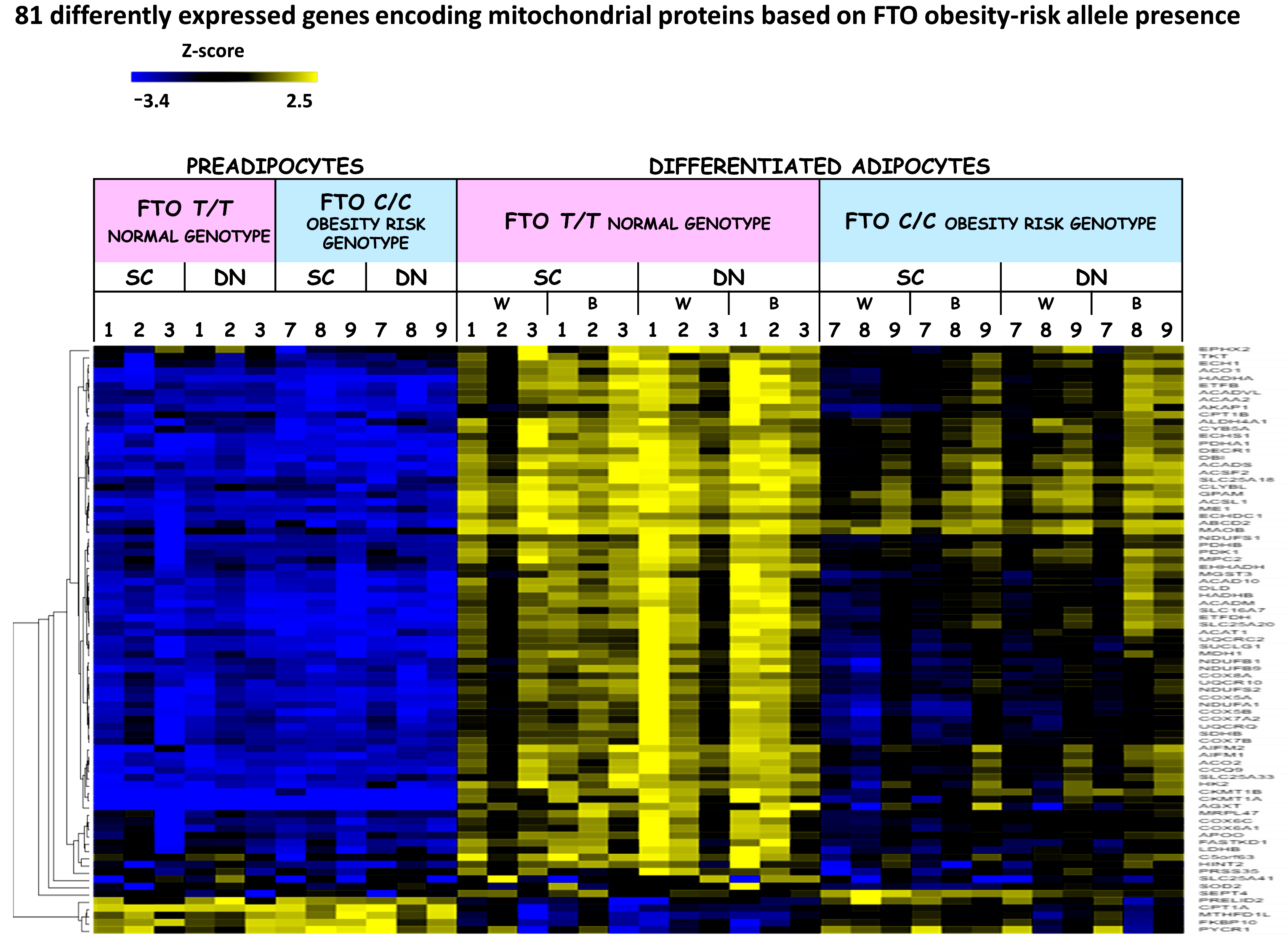

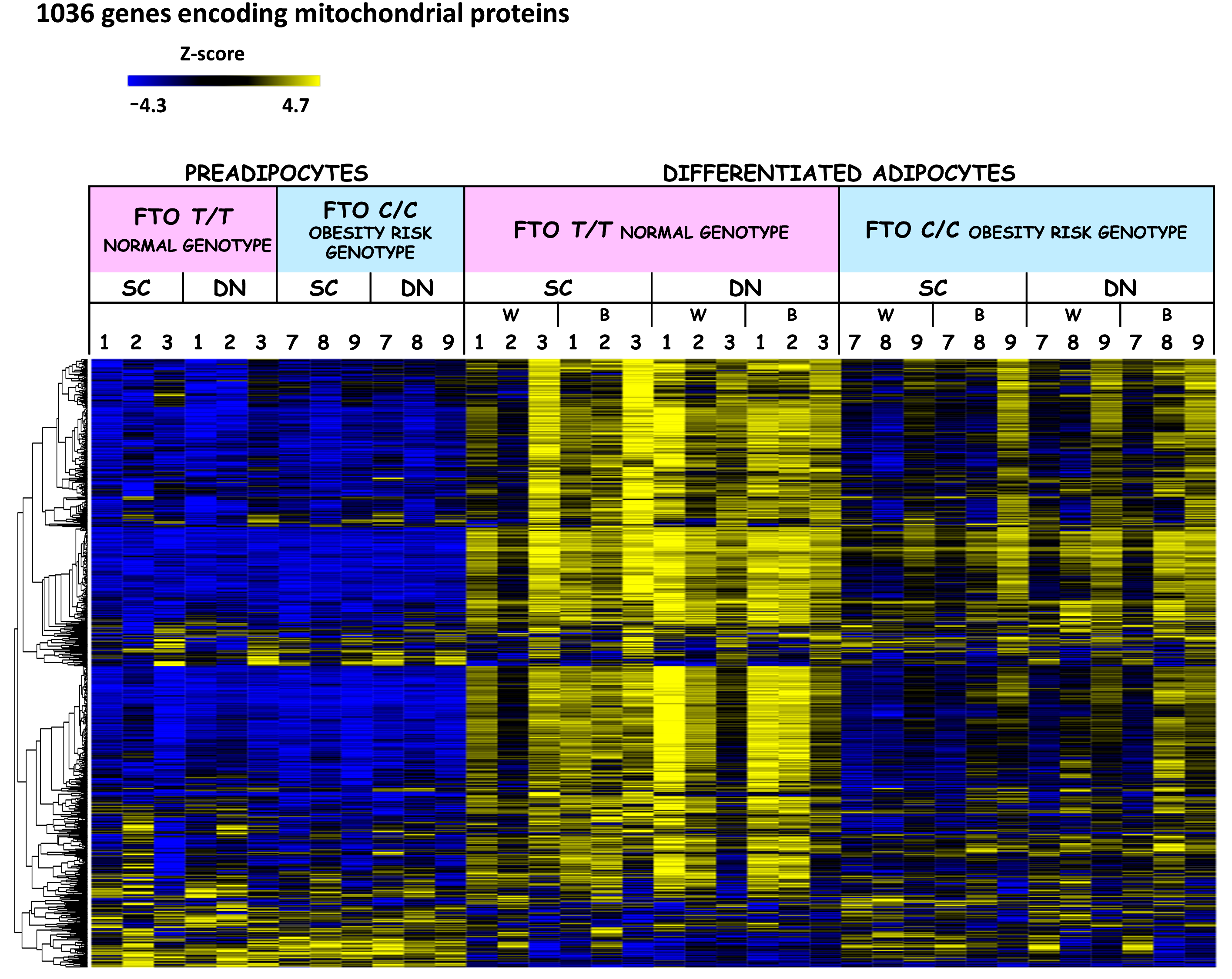


**Brown markers from ProFAT database**

**Brown markers from BATLAS database**

**81 DEGs encoding mitochondrial protein based on the presence of FTO obesity-risk allele**

**1036 mitochondrial protein coding genes based on the presence of FTO obesity-risk allele**

**Supplementary Figure 5.**


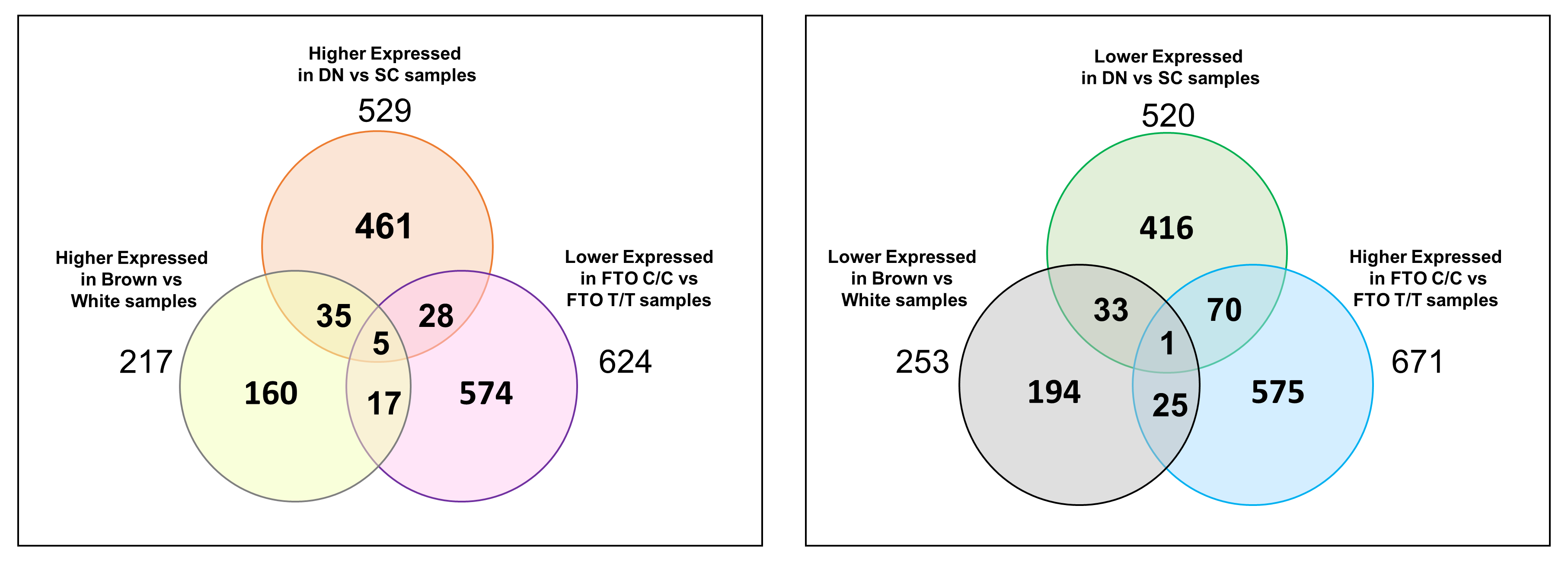


**A**

**B**

**Supplementary Figure 6. Number of differentially expressed genes in differentiated adipocytes based on tissue origin, differentiation protocol and the presence of the FTO obesity-risk allele.** **(A)** Venn diagram shows significantly higher expressed genes number in samples expected higher thermogenic activity, and among of these genes several were lower expressed in FTO obesity-risk genotype samples **(B)** shows significantly lower expressed genes number in samples expected higher thermogenic activity, and among of these genes many expressed higher in FTO obesity-risk genotype samples. SC: subcutaneous; DN: deep-neck.

**Supplementary Figure 6.**
