# Supplementary material for: FTO Intronic SNP Strongly Influences Human Neck Adipocyte Browning Determined by Tissue and PPARγ Specific Regulation: A Transcriptome Analysis
Source: Cells. 2020 Apr 16;9(4):987. doi: 10.3390/cells9040987 (PMC7227023; doi:10.3390/cells9040987)
Supplement: Supplementary file 1 [file cells-09-00987-s001.zip › Supplementary Tables Cells revised.docx]

**Supplementary Table 1.**

| **Indicated cell type or component** | **CD markers** | **Percentage of positive cells (SC PA)** | **Percentage of positive cells (DN PA)** |
| --- | --- | --- | --- |
| Hematopoietic/ Monocyte markers | *CD34* | 0.01±0.01 | 56.31±48.93 |
|  | *CD45* | 0.00±0.00 | 0.00±0.00 |
|  | *CD47* | 96.24±1.36 | 94.36±1.72 |
|  | *CD338 (ABCG2)* | 27.32±1.36 | 35.61±32.46 |
|  | *HLA-DR* | 30.22±52.34 | 28.46±49.29 |
| Endothelial markers | *CD31 (PECAM)* | 0.00±0.00 | 3.43±5.94 |
|  | *CD54 (ICAM-1)* | 37.94±39.11 | 32.79±56.73 |
| MSC/Fibroblast markers | *CD73* | 96.04±1.84 | 94.49±5.59 |
|  | *CD90 (Thy-1)* | 94.62±1.20 | 87.78±6.23 |
|  | *CD105 (Endoglin)* | 56.81±49.23 | 20.53±35.56 |
| Integrins and CAMs | *CD29 (Integrin ß1)* | 87.93±15.62 | 97.27±3.23 |
|  | *CD36* | 76.40±4.32 | 79.22±7.78 |
|  | *CD44 (H-CAM.Hermes)* | 85.31±11.82 | 60.29±48.31 |
|  | *CD49a (Integrin α1)* | 50.50±45.76 | 68.26±4.89 |
|  | *CD49d (Integrin α4)* | 27.67±47.92 | 19.12±33.11 |
|  | *CD146 (MCAM)* | 4.13±6.66 | 0.96±1.67 |
|  | *CD325* | 81.75±13.17 | 90.92±6.46 |

**Supplementary Table 1.** **Analysis of the surface antigen patterns of SC and DN preadipocytes.** Expression of 17 markers was determined in undifferentiated preadipocytes of 3 independent donors with T/C genotype for the rs1421085 locus in the *FTO* gene by flow cytometry. Four groups of markers were tested: hematopoietic/monocyte, endothelial, MSC/fibroblast markers, integrins and CAMs. The numbers represent the percentage of positive cells ±SD. SC: Subcutaneous, DN: Deep-neck, PA: preadipocyte.

**Supplementary Table 2.**

| **GENE ID** | **ENCODED PROTEIN** | **ROLE IN ADIPOGENESIS** |
| --- | --- | --- |
| ***IGF1*** | Insulin-like growth factor I | Differentiation signal |
| ***SOX9*** | Transcription factor SOX-9 | Differentiation signal |
| ***PPARG*** | Peroxisome proliferator-activated receptor gamma | Adipogenic transcription factor |
| ***STAT3*** | Signal transducer and activator of transcription 3 | Differentiation signal |
| ***CEBPA*** | CCAAT/enhancer-binding protein alpha | Adipogenic transcription factor |
| ***SREBF1*** | Sterol regulatory element-binding protein 1 | Lipogenic gene regulators |
| ***SLC2A4*** | Glucose transporter member 4 | Adipocyte protein |
| ***FABP4*** | Fatty acid-binding protein. adipocyte | Adipocyte protein |
| ***LPL*** | Lipoprotein lipase | Adipocyte protein |
| ***AGPAT2*** | 1-acylglycerol-3-phosphate O-acyltransferase 2 | Adipocyte protein |
| ***PLIN1*** | Perilipin-1 | Adipocyte protein |
| ***ADIPOQ*** | Adiponectin | Adipocyte protein |
| ***LEP*** | Leptin | Adipocyte protein |
| ***STAT5A*** | Signal transducer and activator of transcription 5 | Adipogenesis regulator |
| ***EBF1*** | Transcription factor COE1/Early B-factor 1 | Adipogenesis regulator |
| ***CIDEC*** | Cell death inducing DFFA like effector C | Lipid droplet formation |
| ***PPARGC1A*** | Peroxisome proliferator-activated receptor gamma coactivator 1-alpha | Transcriptional co-factor |
| ***PPARGC1B*** | Peroxisome proliferator-activated receptor gamma coactivator 1-beta | Transcriptional co-factor |

**Supplementary Table 2. List of general adipocyte marker genes, the encoded proteins and their role in adipogenesis**

**Supplementary Table 3.**

| **Genes** | **Encoded Protein** | **FC DNW_SCW/DNB_SCB** | **pValue DNW_SCW/DNB_SCB** | **FC DNB_DNW/SCB_SCW** | **pValue DNB_DNW/SCB_SCW** |
| --- | --- | --- | --- | --- | --- |
| **GROUP 1** | | | | | |
| ***AC007938.2*** |  | -/2.6 | -/0.02 | 2.6/- | 0.03/- |
| ***AC009126.1*** |  | -/1.8 | -/0.009 | 2.3/- | 0.0003/- |
| ***AC034102.6*** |  | -/2.3 | -/0.01 | 2.3/- | 0.02/- |
| ***AL606534.4*** |  | 3.6/3.2 | 0.003/0.005 | 2.7/2.9 | 0.04/0.03 |
| ***ARNTL2*** | Aryl hydrocarbon receptor nuclear translocator-like protein 2 | 2.1/2.3 | 0.001/4.2E-05 | 2.2/1.9 | 9.5E-04/1.5E-02 |
| ***B3GNT5*** | Lactosylceramide 1,3-N-acetyl-beta-D-glucosaminyltransferase | 2.2/1.9 | 0.009/0.04 | 2.2/2.6 | 0.009/0.002 |
| ***CIDEA*** | Cell death activator CIDE-A | 3.6/- | 0.03/- | 3.8/7.9 | 0.02/0.0001 |
| ***CKMT1B*** | Creatine kinase 1B | 3.1/- |  | -/2.6 | -/0.04 |
| ***CPA2*** | Carboxypeptidase A2 | 2.9/- | 0.01/- | 3.5/4.9 | 0.0004/8.5E-05 |
| ***CPT1B*** | Carnitine O-palmitoyltransferase 1, muscle isoform | 1.8/2.1 | 0.015/0.0001 | 2.3/2.0 | 4.6E-05/8.2E-03 |
| ***DAZL*** | Deleted in azoospermia-like | -/3.9 | -/0.003 | 3.6/- | 0.01/- |
| ***DRP2*** | Dystrophin-related protein 2 | -/2.3 | -/0.007 | 2.4/- | 0.005/- |
| ***FAM151A*** | Protein FAM151A | 4.5/2.9 | 1.2E-05/0.002 | 2.4/3.7 | 0.03/0.0007 |
| ***FAM189A2*** | Protein FAM189A2 | -/2.6 | -/0.006 | 2.3/- | 0.03/- |
| ***GPR160*** | Probable G-protein coupled receptor 160 | -/2.5 | -/0.0003 | 2.4/- | 0.001/- |
| ***KCNIP2*** | Kv channel-interacting protein 2 | /2.4 | /0.02 | 2.7/2.3 | 0.008/0.03 |
| ***KCNIP2-AS1*** |  | -/2.5 | -/0.01 | 2.5/- | 0.01/- |
| ***LINC01347*** |  | 3.1/2.7 | 0.0007/0.01 | 2.6 | 0.05 |
| ***LINC02458*** |  | -/2.6 | -/0.03 | 2.9/- | 0.01/- |
| ***OASL*** | 2'-5'-oligoadenylate synthase-like protein | 4.1/- | 0.006/- | 5.2/11.0 | 0.001/1.0E-06 |
| ***PCDH19*** |  | 2.7/4.7 | 0.05/0.0001 | 4.4/- | 0.0005/- |
| ***PM20D1*** | N-fatty-acyl-amino acid synthase | 4.5/4.2 | 0.003/0.006 | 7.0/7.5 | 9.8E-05/5.8E-05 |
| ***RASD1*** | Dexamethasone-induced Ras-related protein 1 | 2.04/- | 0.02/- | 2.6/3.4 | 1.7E-03/2.6E-05 |
| ***RASSF6*** | Ras association domain-containing protein 6 | -/2.7 | -/0.03 | 2.9/- | 0.04/- |
| ***RGL3*** | Ral guanine nucleotide dissociation stimulator-like 3 | 2,4/- | 0.01/- | 2.3/3.1 | 2.8E-02/1.5E-03 |
| ***RXRG*** | Retinoic acid receptor RXR-gamma | 2.7/- | 0.02/- | -/2.8 | -/0.02 |
| ***SCN4A*** | Sodium channel protein type 4 subunit alpha | 3.7/- | 2.3E-05/- | 2.8/6.3 | 1.3E-03/5.7E-09 |
| ***SLC19A3*** | Thiamine transporter 2 | 1.9/- | 0.028/- | 2.0/2.1 | 4.6E-02/2.2E-02 |
| ***SLC7A10*** | Amino acids transporter | 5.3/4.3 | 2.6E-05/0.003 | 2.7/3.3 | 5.0E-02/1.6E-02 |
| **GROUP 2** | | | | | |
| ***CPA4*** | Carboxypeptidase A4 | 2.4/2.1 | 0.03/0.04 | 4.8/5.3 | 1.4E-06/1.1E-06 |
| ***CX3CL1*** | Fractalkine | 4.8/- | 0.004/- | -/3.7 | -/4.0E-02 |
| ***CXCL16*** | C-X-C motif chemokine 16 | 1.9/- | 0.0008/- | -/1.9 | -/3.0E-03 |
| ***EGFLAM*** | Pikachurin | 2.1/- | 0.0004 | 1.9/2.4 | 0.009/0.0001 |
| ***EPB41L4B*** | Band 4.1-like protein 4B | 2.1/- | 0.02/- | 2.3/2.6 | 0.01/0.002 |
| ***LINC00623*** |  | 2.4/- | 0.02/- | 2.6 | 2.0E-02 |
| ***MEST*** | Mesoderm-specific transcript homolog protein | 2.6/2.2 | 1.3E-05/0.0009 | 2.5/3.0 | 0.0001/2.6E-06 |
| ***MYOM1*** | Myomesin-1 | 2.0/- | 0.004/- | 1.9 | 1.0E-02 |
| ***SLC22A11*** | Solute carrier family 22 member 11 | -/3.7 | -/0.01 | 5.6/- | 0.002/- |
| ***SLCO2B1*** | organic anion transporter family member 2B1 | -/2.5 | -/0.03 | 3.1/- | 0.006/- |
| ***TSLP*** | Thymic stromal lymphopoietin | 2.5/2.3 | 0.002/0.002 | 2.5/2.7 | 0.0006/0.0007 |

**Supplementary Table 3. Common genes between anatomical location comparison and upregulated after brown differentiation protocol,** FC: fold change

**Supplementary Table 4.**

| **Genes** | **Encoded Protein** | **FC DNW_SCW/DNB_SCB** | **pValue DNW_SCW/DNB_SCB** | **FC DNW_DNB/SCW_SCB** | **pValue DNW_DNB/SCW_SCB** |
| --- | --- | --- | --- | --- | --- |
| **GROUP 3** | | | | | |
| ***AL049825.1*** |  | 3.6 | 0.04 | 4.6/- | 0.01/- |
| ***ANO4*** | Anoctamin-4 | 2.7/- | 0.006/- | 2.3 | 0.04 |
| ***CCDC146*** | Coiled-coil domain-containing protein 146 | 1.9/- | 2.6E-05/- | 1.8 | 0.0005 |
| ***CCNA1*** | Cyclin-A1 | 3.4 | 0.02 | 5.0/- | 0.002/- |
| ***DIO2*** | Type II iodothyronine deiodinase | 3.5/- | 0.004/- | 2.8 | 0.04 |
| ***FBN2*** | Fibrillin-2 | 5.9/6.7 | 3.9E-07/6.1E-08 | 2.6/- | 0.03/- |
| ***GPC3*** | Glypican-3 | 2.9/- | 0.0002/- | 2.9 | 0.001 |
| ***GPM6B*** | Neuronal membrane glycoprotein M6-b | 3.5/- | 5.3E-05/- | 3.2 | 0.0005 |
| ***IQCH-AS1*** |  | 1.9/- | 0.002/- | 1.8 | 0.01 |
| ***KLF5*** | Krueppel-like factor 5 | 3.1/2.3 | 2.0E-05/0.005 | 1.9 | 0.04 |
| ***LINC00242*** |  | 2.1/- | 0.04/- | 3.2 | 0.0009 |
| ***LINC00702*** |  | 2.6/3.3 | 0.001/0.0002 | 3.9/2.9 | 0.0003 |
| ***LINC01238*** |  | 5.6/- | 0.0007/- | 4.9 | 0.005 |
| ***LINC02268*** |  | 5.8 | 0.009 | 2.6 | 0.05 |
| ***MARCO*** | Macrophage receptor MARCO | 3.6/- | 0.005/- | 3.1 | 0.03 |
| ***MOB3B*** | MOB kinase activator 3B | 2.6/2.1 | 0.001/0.03 | 2.2 | 0.02 |
| ***PPIAP39*** |  | 2.7 | 0.005 | 3.4/2.4 | 0.0007/0.02 |
| ***ROBO2*** | Roundabout homolog 2 | 2.6/- | 0.02/- | 4 | 0.0005 |
| ***SLITRK1*** | SLIT and NTRK-like protein 1 | 6.5/6.3 | 2.7E-05/0.0002 | 3.4 | 0.02 |
| ***SSPO*** | SCO-spondin | 2.6/2.4 | 6E-05/0.001 | 1.8 | 0.04 |
| ***TRIM55*** | Tripartite motif-containing protein 55 | 2.9/- | 0.02/- | 3.3 | 0.02 |
| ***TRNP1*** | TMF-regulated nuclear protein 1 | 1.9/2.0 | 0.02/0.01 | 2.6/2.4 | 0.0003/0.0009 |
| ***TRPV6*** | Transient receptor potential cation channel subfamily V6 | 3.0/- | 0.03/- | 5 | 0.003 |
| ***ZNF385B*** | Zinc finger protein 385B | 3.2 | 0.04 | 6.7/3.3 | 0.0002/0.02 |
| **GROUP 4** | | | | | |
| ***AL096865.1*** |  | 4.5/9.2 | 0.0004/0.0002 | 4.9/- | 0.040 |
| ***COL8A1*** | Collagen alpha-1(VIII) chain | 3.4/2.8 | 0.0003/0.005 | /2.5 | 0.030 |
| ***CRISPLD2*** | Cysteine-rich secretory protein LCCL domain-containing 2 | 2.5 | 0.04 | /3.7 | 0.001 |
| ***DPT*** | Dermatopontin | 2.4/2.5 | 0.01/0.01 | 2.6/2.6 | 0.012/0.01 |
| ***GRIA1*** | Glutamate receptor 1 | 3.5 | 0.04 | /4.4 | 1.5E-02 |
| ***NEGR1*** | Neuronal growth regulator 1 | 1.8/- | 0.02/- | 2.9/2.7 | 5E-05/1.1E-05 |
| ***RSPO1*** | R-spondin-1 | 4.6/6.4 | 2.7E-05/0.2E-06 | 2.6/3.5 | 0.006/0.04 |
| ***TNC*** | Tenascin | 2.8/3.9 | 0.03/0.002 | /3.3 | 0.02/- |
| ***TRPC6*** | Short transient receptor potential channel 6 | 19.7/7.05 | 5.0E-15/1.5E-05 | 4.3/- | 7.0E-04 |
| ***ZNF467*** | Zinc finger protein 467 | 2.3/2.3 | 0.009/0.02 | 2.1 | 4.0E-02 |

**Supplementary Table 4. Common genes between anatomical location comparison and downregulated after brown differentiation protocol,** FC: fold change

**Supplementary Table 5A.**

| **KEGG enriched pathways in FTO obese LOW expressed genes** | **KEGG enriched pathways in BATLAS** | | **KEGG enriched pathways in ProFAT** |
| --- | --- | --- | --- |
| ***Metabolic pathways*** | **Metabolic pathways** | | *Metabolic pathways* |
| ***Fatty acid metabolism*** | **Thermogenesis** | | *Parkinson's disease* |
| ***Thermogenesis*** | **Oxidative phosphorylation** | | *Huntington's disease* |
| ***Oxidative phosphorylation*** | **Parkinson's disease** | | *Thermogenesis* |
| ***Carbon metabolism*** | **Huntington's disease** | | *Non-alcoholic fatty liver disease (NAFLD)* |
| ***Huntington's disease*** | **Non-alcoholic fatty liver disease (NAFLD)** | | *Carbon metabolism* |
| ***Parkinson's disease*** | **Alzheimer's disease** | | *Alzheimer's disease* |
| ***Fatty acid degradation*** | **Carbon metabolism** | | *Oxidative phosphorylation* |
| ***Alzheimer's disease*** | **Citrate cycle (TCA cycle)** | | *Citrate cycle (TCA cycle)* |
| ***Non-alcoholic fatty liver disease (NAFLD)*** | **Fatty acid degradation** | | *2-Oxocarboxylic acid metabolism* |
| ***Valine. leucine and isoleucine degradation*** | **Fatty acid metabolism** | | *Fatty acid metabolism* |
| ***Citrate cycle (TCA cycle)*** | **Valine. leucine and isoleucine degradation** | | *Glyoxylate and dicarboxylate metabolism* |
| ***PPAR signaling pathway*** | **Cardiac muscle contraction** | | *Fatty acid degradation* |
| ***Fatty acid elongation*** | **PPAR signaling pathway** | | *Valine. leucine and isoleucine degradation* |
| ***Pyruvate metabolism*** | **2-Oxocarboxylic acid metabolism** | | *Glycolysis / Gluconeogenesis* |
| ***Cardiac muscle contraction*** | **Pyruvate metabolism** | | *Fatty acid elongation* |
| ***Glyoxylate and dicarboxylate metabolism*** | **Glyoxylate and dicarboxylate metabolism** | | *Cardiac muscle contraction* |
| ***Glycolysis / Gluconeogenesis*** | **Glycolysis / Gluconeogenesis** | | *Pyruvate metabolism* |
| ***2-Oxocarboxylic acid metabolism*** | **Fatty acid elongation** | | *PPAR signaling pathway* |
| **Propanoate metabolism** | **Propanoate metabolism** | | Biosynthesis of amino acids |
| **Peroxisome** | **Butanoate metabolism** | | Retrograde endocannabinoid signaling |
| **Butanoate metabolism** | **Tryptophan metabolism** | | Central carbon metabolism in cancer |
| **Adipocytokine signaling pathway** | **Peroxisome** | | Glycine. serine and threonine metabolism |
| **Tryptophan metabolism** | **beta-Alanine metabolism** | |  |
| **beta-Alanine metabolism** | **Adipocytokine signaling pathway** | | |
| Biosynthesis of unsaturated fatty acids | Retrograde endocannabinoid signaling | | |
| Type I diabetes mellitus | Lysine degradation | |  |
| AMPK signaling pathway | Biosynthesis of amino acids | | |
| Glucagon signaling pathway | Ribosome | |  |
| Insulin resistance |  | |  |
| Allograft rejection |  | |  |
| Steroid hormone biosynthesis | | |  |
| Graft-versus-host disease | |  |  |

**Supplementary Table 5B.**

| **REACTOM enriched pathways in FTO obese LOW expressed genes** | **REACTOM enriched pathways in BATLAS** | | | | **REACTOM enriched pathways in ProFAT** | |  |
| --- | --- | --- | --- | --- | --- | --- | --- |
| ***Metabolism*** | **The citric acid (TCA) cycle and respiratory electron transport** | | | | *The citric acid (TCA) cycle and respiratory electron transport* | |  |
| ***Metabolism of lipids*** | **Metabolism** | | | | *Metabolism* | |  |
| ***The citric acid (TCA) cycle and respiratory electron transport*** | **Respiratory electron transport** | | | | *Respiratory electron transport, ATP synthesis by chemiosmotic coupling, and heat production by uncoupling proteins.* | |  |
| ***Fatty acid metabolism*** | **Respiratory electron transport, ATP synthesis by chemiosmotic coupling, and heat production by uncoupling proteins.** | | | | *Respiratory electron transport* | |  |
| ***Respiratory electron transport, ATP synthesis by chemiosmotic coupling, and heat production by uncoupling proteins.*** | **Pyruvate metabolism and Citric Acid (TCA) cycle** | | | | *Pyruvate metabolism and Citric Acid (TCA) cycle* | |  |
| ***Respiratory electron transport*** | **Citric acid cycle (TCA cycle)** | | | | *Citric acid cycle (TCA cycle)* | |  |
| ***Mitochondrial Fatty Acid Beta-Oxidation*** | **Fatty acid metabolism** | | | | *Mitochondrial Fatty Acid Beta-Oxidation* | |  |
| ***Pyruvate metabolism and Citric Acid (TCA) cycle*** | **Mitochondrial Fatty Acid Beta-Oxidation** | | | | *Fatty acid metabolism* | |  |
| ***Signaling by Retinoic Acid*** | **Glyoxylate metabolism and glycine degradation** | | | | *Glyoxylate metabolism and glycine degradation* | |  |
| ***mitochondrial fatty acid beta-oxidation of saturated fatty acids*** | **Metabolism of lipids** | | | | *mitochondrial fatty acid beta-oxidation of saturated fatty acids* | |  |
| ***Beta oxidation of hexanoyl-CoA to butanoyl-CoA*** | **mitochondrial fatty acid beta-oxidation of saturated fatty acids** | | | | *Regulation of pyruvate dehydrogenase (PDH) complex* | |  |
| ***Beta oxidation of decanoyl-CoA to octanoyl-CoA-CoA*** | **Beta oxidation of hexanoyl-CoA to butanoyl-CoA** | | | | *Beta oxidation of hexanoyl-CoA to butanoyl-CoA* | |  |
| ***Regulation of pyruvate dehydrogenase (PDH) complex*** | **Beta oxidation of decanoyl-CoA to octanoyl-CoA-CoA** | | | | *Beta oxidation of decanoyl-CoA to octanoyl-CoA-CoA* | |  |
| ***Glyoxylate metabolism and glycine degradation*** | **Signaling by Retinoic Acid** | | | | *Signaling by Retinoic Acid* | |  |
| ***Citric acid cycle (TCA cycle)*** | **Regulation of pyruvate dehydrogenase (PDH) complex** | | | | *Metabolism of lipids* | |  |
| *Import of palmitoyl-CoA into the mitochondrial matrix* | **Protein localization** | | | | *Import of palmitoyl-CoA into the mitochondrial matrix* | |  |
| **Pyruvate metabolism** | **Mitochondrial biogenesis** | | | | Complex I biogenesis | |  |
| **Beta oxidation of octanoyl-CoA to hexanoyl-CoA** | **Cristae formation** | | | | Mitochondrial protein import | |  |
| **Mitochondrial biogenesis** | **Beta oxidation of palmitoyl-CoA to myristoyl-CoA** | | | | Transcriptional activation of mitochondrial biogenesis | |  |
| **Beta oxidation of palmitoyl-CoA to myristoyl-CoA** | **Beta oxidation of lauroyl-CoA to decanoyl-CoA-CoA** | | | | Lysine catabolism | |  |
| **Beta oxidation of lauroyl-CoA to decanoyl-CoA-CoA** | **Beta oxidation of octanoyl-CoA to hexanoyl-CoA** | | | | Branched-chain amino acid catabolism | |  |
| **Cristae formation** | **Pyruvate metabolism** | | | | Metabolism of amino acids and derivatives | |  |
| **Peroxisomal protein import** | **Peroxisomal protein import** | | | |  |  |  |
| **Peroxisomal lipid metabolism** | **Peroxisomal lipid metabolism** | | | |  |  |  |
| **Protein localization** | Complex I biogenesis | | | |  |  |  |
| Endosomal/Vacuolar pathway | Mitochondrial translation initiation | | | |  |  |  |
| Signaling by Nuclear Receptors | Mitochondrial translation elongation | | | |  |  |  |
| Triglyceride metabolism | Mitochondrial translation termination | | | |  |  |  |
| mitochondrial fatty acid beta-oxidation of unsaturated fatty acids | Mitochondrial protein import | | | |  |  |  |
| Synthesis of very long-chain fatty acyl-CoAs | Lysine catabolism | | | |  |  |  |
| Antigen Presentation: Folding, assembly and peptide loading of class I MHC | Branched-chain amino acid catabolism | | | |  |  |  |
| Glycerophospholipid biosynthesis | Metabolism of amino acids and derivatives | | | |  |  |  |
| Synthesis of bile acids and bile salts via 24-hydroxycholesterol | Beta oxidation of butanoyl-CoA to acetyl-CoA | | | |  |  |  |
| Synthesis of bile acids and bile salts via 27-hydroxycholesterol | Gluconeogenesis | | | |  |  |  |
| Interferon gamma signaling | Mitochondrial calcium ion transport | | | |  |  |  |
| Acyl chain remodeling of CL | | | | |  |  |  |
| Formation of ATP by chemiosmotic coupling |  | | | |  |  |  |
| Metabolism of steroids |  |  | | |  |  |  |
| Linoleic acid (LA) metabolism | | | | |  |  |  |
| RA biosynthesis pathway |  |  | | |  |  |  |
| Synthesis of bile acids and bile salts via 7alpha-hydroxycholesterol |  |  |  |  |  |  |  |
| TP53 Regulates Metabolic Genes | | | | |  |  |  |
| Triglyceride catabolism |  |  | | |  |  |  |
| PPARA activates gene expression | | | | |  |  |  |
| Retinoid metabolism and transport | | | | |  |  |  |
| alpha-linolenic acid (ALA) metabolism | | | | |  |  |  |
| Antigen processing-Cross presentation | | | | |  |  |  |
| Phospholipid metabolism |  |  | | |  |  |  |
| Visual phototransduction |  |  | | |  |  |  |
| Triglyceride biosynthesis |  |  | | |  |  |  |
| Beta oxidation of myristoyl-CoA to lauroyl-CoA | | |  |  |  |  |  |
| Immunoregulatory interactions between a Lymphoid and a non-Lymphoid cell | | | | | | | |

**Supplementary Table 5. Tables show the significantly enriched pathways identified by genes expressed lower in *FTO* C/C samples, BATLAS and ProFAT gene set. (A) KEGG pathways (B) REACTOM pathways.** Red letters: Pathways presented in all three samples; Bold: Common Pathways in *FTO* obesity-risk based DEGs and BATLAS; Italic font: Common Pathways in *FTO* obesity-risk based DEGs and ProFAT. Blue letters: Common Pathways only in BATLAS and ProFAT.

**Supplementary Table 6A**.

| **Gene** | **BC** **score** | **Bridges** | **FC** | **Gene Description** |
| --- | --- | --- | --- | --- |
| ***ATP5B*** | 2015 | 18 | 2.2 | ATP synthase beta polypeptide |
| ***SDHB*** | 1028 | 18 | 2.0 | succinate dehydrogenase complex |
| ***SUCLG1*** | 1064 | 16 | 2.1 | succinate-CoA ligase |
| ***SOD2*** | 2418 | 14 | 2.4 | superoxide dismutase 2 |
| ***DECR1*** | 7717 | 13 | 1.8 | 2,4-dienoyl CoA reductase 1 |
| ***CD36*** | 1730 | 13 | 2.4 | Thrombospondin Receptor |
| ***ACADM*** | 1531 | 13 | 2.2 | acyl-CoA dehydrogenase |
| ***NDUFS1*** | 200 | 13 | 1.8 | NADH dehydrogenase Fe-S protein 1 |
| ***MDH1*** | 968 | 12 | 2.7 | malate dehydrogenase 1 |
| ***PPARGC1A*** | 854 | 12 | 2.6 | peroxisome proliferator-activated receptor gamma |
| ***UQCRC2*** | 139 | 12 | 2.0 | ubiquinol-cytochrome c reductase core protein II |
| ***ACSL1*** | 525 | 11 | 1.8 | acyl-CoA synthetase long-chain family member 1 |
| ***CPT1B*** | 294 | 11 | 2.0 | carnitine palmitoyltransferase 1B |
| ***CCL2*** | 4845 | 10 | 2.5 | chemokine (C-C motif) ligand 2 |
| ***LIPE*** | 3792 | 10 | 2.6 | lipase hormon sensitive |

**Supplementary Table 6B.**

| **Gene** | **BC** **score** | **Bridges** | **FC** | **Gene Description** |
| --- | --- | --- | --- | --- |
| ***FN1*** | 17619 | 37 | 2.1 | fibronectin 1 |
| ***CTGF*** | 5508 | 16 | 2.6 | connective tissue growth factor |
| ***VCAN*** | 1117 | 16 | 2.4 | versican |
| ***TGFB1*** | 5194 | 14 | 1.9 | transforming growth factor, beta 1 |
| ***COL1A1*** | 4343 | 14 | 2.7 | collagen, type I, alpha 1 |
| ***CDH2*** | 3971 | 14 | 2.6 | cadherin 2, type 1, N-cadherin (neuronal) |
| ***BDNF*** | 5515 | 13 | 2.0 | brain-derived neurotrophic factor |
| ***VCAM1*** | 3666 | 12 | 3.1 | vascular cell adhesion molecule 1 |
| ***TIMP1*** | 2592 | 12 | 2.5 | TIMP metallopeptidase inhibitor 1 |
| ***LOX*** | 1377 | 12 | 2.0 | lysyl oxidase |
| ***VEGFC*** | 3627 | 11 | 1.9 | vascular endothelial growth factor C |
| ***FOS*** | 2986 | 9 | 2.0 | FBJ murine osteosarcoma viral oncogene homolog |
| ***SDC1*** | 2729 | 9 | 2.4 | syndecan 1 |
| ***BGN*** | 1644 | 9 | 3.9 | biglycan |
| ***GLI1*** | 5192 | 8 | 2.7 | GLI family zinc finger 1 |

**SupplementaryTable 6. Betweenness centrality (BC) score and Bridges number identified based on STRING Interactome network and Fold Changes (FC) identified by DESeq R of the differentially expressed genes based on the presence of the FTO obesity risk allele*.*** Analyses of the lower expressed genes **(A)** and the higher expressed genes **(B)** in FTO obesity-risk samples, n=6 donors

**Supplementary Table 7.**

| **Genes less expressed in FTO C/C and high in DN samples** | **FC** | **TRANSCRIPT** |  |
| --- | --- | --- | --- |
| ***CKMT1A*** | 4.9 | creatine kinase, mitochondrial 1A | |
| ***LINC01348*** | 4.6 | Long Intergenic Non-Protein Coding RNA, nearest gene TOMM20 | |
| ***PRSS35*** | 4.3 | Protease, serine35 | |
| ***SPTA1*** | 3.6 | Spectrin, alpha, erythrocytic 1 (elliptocytosis 2) | |
| ***EPB42*** | 3.5 | erythrocyte membrane protein band 4.2 | |
| ***ZDHHC19*** | 3.2 | zinc finger, DHHC-type containing 19 | |
| ***SNCG*** | 3.1 | Synuclein, gamma (breast cancer-specific protein 1) | |
| ***HYAL1*** | 3.0 | hyaluronoglucosaminidase 1 | |
| ***EMB*** | 3.0 | embigin | |
| ***LINC02458*** | 2.8 | Long Intergenic Non-Protein Coding RNA, nearest gene AC006199.1 pseudogene | |
| ***CKMT1B*** | 2.8 | creatine kinase, mitochondrial 1B | |
| ***GBP1P1*** | 2.8 | guanylate binding protein 1, interferon-inducible pseudogene 1 | |
| ***LINC01914*** | 2.8 | Long Intergenic Non-Protein Coding RNA, nearest gene c2orf91 | |
| ***RHOXF1-AS1*** | 2.7 | Rhox Homebox family Member 1 antisense RNA | |
| ***SGK2*** | 2.5 | serum/glucocorticoid regulated kinase 2 | |
| ***FABP3*** | 2.5 | fatty acid binding protein 3, muscle and heart (mammary-derived growth inhibitor) | |
| ***C1QTNF1*** | 2.5 | C1q and tumor necrosis factor related protein 1 | |
| ***RPS6KL1*** | 2.5 | ribosomal protein S6 kinase-like 1 | |
| ***AKR1C8P*** | 2.5 | Aldo-Keto Reductase Family 1 Member C8, Pseudogene | |
| ***SOD2*** | 2.4 | superoxide dismutase 2, mitochondrial | |
| ***PLA2G4A*** | 2.4 | phospholipase A2, group IVA (cytosolic. calcium-dependent) | |
| ***FAM189A2*** | 2.3 | family with sequence similarity 189, member A2 | |
| ***GPLD1*** | 2.2 | glycosylphosphatidylinositol specific phospholipase D1 | |
| ***SLC19A3*** | 2.1 | solute carrier family 19 (thiamine transporter), member 3 | |
| ***CPT1B*** | 2.0 | carnitine palmitoyltransferase 1B (muscle) | |
| ***TPRG1*** | 2.0 | tumor protein p63 regulated 1 | |
| ***LINC01140*** | 2.0 | LINC- RNA, complement to AC093155.3 protein coding transcript, Long Intergenic Non-Protein Coding RNA | |
| ***RNF207*** | 1.9 | ring finger protein 207 | |
| ***AC010319.5*** | 1.9 | LINC-RNA, complement to AC010319.2 protein coding transcript, nearest genes TMEM221. NXNL1 | |
| ***GCKR*** | 1.9 | glucokinase (hexokinase 4) regulator | |
| ***EHHADH*** | 1.9 | enoyl-CoA, hydratase/3-hydroxyacyl CoA dehydrogenase | |
| ***AKAP1*** | 1.9 | A kinase (PRKA) anchor protein 1 | |
| ***CRYAB*** | 1.8 | crystallin. alpha B | |

**Supplementary Table 7. Genes that are significantly less expressed in the FTO obesity-risk genotypes (C/C) as compared to normal (T/T) and higher in DN as compared to SC samples.** FC: fold change after white and brown differentiation, average fold change (FC) when two data considered.

**Supplementary Table 8.**

| **Genes highly expressed in FTO C/C and low in DN samples** | **FC** | **TRANSCRIPT** |
| --- | --- | --- |
| ***HAPLN1*** | 8.9 | hyaluronan and proteoglycan link protein 1 |
| ***CMKLR1*** | 7.8 | chemokine-like receptor 1 |
| ***CADPS*** | 6.0 | Ca++-dependent secretion activator |
| ***PTPRD-AS1*** | 5.4 | Protein Tyrosine Phosphatase Receptor Type D antisense 1 |
| ***IL11*** | 5.0 | interleukin 11 |
| ***COL13A1*** | 4.7 | Collagen, type XIII. alpha 1 |
| ***ACAN*** | 4.7 | aggrecan |
| ***AC244153.1*** | 4.5 | novel transcript, lncRNA antisense to ARHGAP23 (Rho GTPase Activating Protein 23) |
| ***COL8A2*** | 4.4 | Collagen, type VIII. alpha 2 |
| ***OPCML*** | 4.2 | opioid binding protein/cell adhesion molecule-like |
| ***NRG1*** | 4.1 | neuregulin 1 |
| ***LINC00900*** | 4.1 | long intergenic non-protein coding RNA 900 |
| ***EGR2*** | 3.9 | early growth response 2 |
| ***F2RL2*** | 3.9 | coagulation factor II (thrombin) receptor-like 2 |
| ***TENM2*** | 3.9 | teneurin transmembrane protein 2 |
| ***PCDH10*** | 3.9 | protocadherin 10 |
| ***CLEC2L*** | 3.8 | C-type lectin domain family 2, member L |
| ***IL27RA*** | 3.8 | interleukin 27 receptor, alpha |
| ***COL8A1*** | 3.7 | Collagen, type VIII. alpha 1 |
| ***MYH1*** | 3.6 | Myosin, heavy chain 1, skeletal muscle, adult |
| ***ULBP1*** | 3.5 | UL16 binding protein 1 |
| ***AC114284.1*** | 3.4 | novel transcript, lncRNA |
| ***ITGA11*** | 3.3 | Integrin, alpha 11 |
| ***AC079336.5*** | 3.3 | Novel Transcript, Antisense MYO1D |
| ***GDNF*** | 3.1 | glial cell derived neurotrophic factor |
| ***CDH6*** | 3.0 | cadherin 6, type 2, K-cadherin (fetal kidney) |
| ***HS3ST3A1*** | 2.9 | heparan sulfate (glucosamine) 3-O-sulfotransferase 3A1 |
| ***CASS4*** | 2.9 | Cas scaffolding protein family member 4 |
| ***F2R*** | 2.9 | coagulation factor II (thrombin) receptor |
| ***GDF6*** | 2.9 | growth differentiation factor 6 |
| ***PODNL1*** | 2.9 | podocan-like 1 |
| ***HCN1*** | 2.8 | hyperpolarization activated cyclic nucleotide-gated potassium channel 1 |
| ***COL1A1*** | 2.7 | Collagen, type I. alpha 1 |
| ***EVA1A*** | 2.7 | eva-1 homolog A (C. elegans) |
| ***SHANK1*** | 2.7 | SH3 and multiple ankyrin repeat domains 1 |
| ***EPHB3*** | 2.6 | EPH receptor B3 |
| ***PRR16*** | 2.6 | proline rich 16 |
| ***PRSS12*** | 2.6 | Protease, serine 12 (neurotrypsin, motopsin) |
| ***SEPT4*** | 2.5 | Septin 4 |
| ***HACD4*** | 2.4 | 3-Hydroxyacyl-CoA Dehydratase 4 |
| ***RASGRP2*** | 2.4 | RAS guanyl releasing protein 2 (calcium and DAG-regulated) |
| ***NCKAP5*** | 2.4 | NCK-associated protein 5 |
| ***STAC*** | 2.4 | SH3 and cysteine rich domain |
| ***SDC1*** | 2.4 | syndecan 1 |
| ***KCNG1*** | 2.3 | potassium voltage-gated channel, subfamily G. member 1 |
| ***GDNF-AS1*** | 2.3 | GDNF antisense RNA 1 (head to head) |
| ***CDH11*** | 2.3 | cadherin 11, type 2, OB-cadherin (osteoblast) |
| ***ALDH3B1*** | 2.3 | aldehyde dehydrogenase 3 family, member B1 |
| ***FOXL1*** | 2.3 | forkhead box L1 |
| ***HOXC8*** | 2.3 | homeobox C8 |
| ***FAM86GP*** | 2.2 | family with sequence similarity 86, member G. pseudogene |
| ***TBX5*** | 2.2 | T-box 5 |
| ***MYO1D*** | 2.2 | myosin ID |
| ***FZD7*** | 2.1 | frizzled family receptor 7 |
| ***TGFB1I1*** | 2.1 | transforming growth factor beta 1 induced transcript 1 |
| ***IL20RA*** | 2.1 | interleukin 20 receptor, alpha |
| ***MRC2*** | 2.1 | mannose receptor, C type 2 |
| ***FSCN1*** | 2.1 | fascin homolog 1, actin-bundling protein (Strongylocentrotus purpuratus) |
| ***ACVR2A*** | 2.1 | activin A receptor, type IIA |
| ***BAIAP2L1*** | 2.1 | BAI1-associated protein 2-like 1 |
| ***LMO7*** | 2.1 | LIM domain 7 |
| ***ASNS*** | 2.1 | asparagine synthetase (glutamine-hydrolyzing) |
| ***GALNT5*** | 2.1 | UDP-N-acetyl-alpha-D-galactosamine:polypeptide N-acetylgalactosaminyltransferase 5 (GalNAc-T5) |
| ***SLIT2*** | 2.1 | slit homolog 2 (Drosophila) |
| ***STARD4-AS1*** | 2.0 | StAR Related Lipid Transfer Domain Containing 4 antisense RNA 1 |
| ***IRX3*** | 1.9 | iroquois homeobox 3 |
| ***RUBCNL*** | 1.9 | Rubicon Like Autophagy Enhancer |
| ***TBX5-AS1*** | 1.9 | TBX5 antisense RNA 1 |
| ***SPON1*** | 1.8 | spondin 1, extracellular matrix protein |
| ***WNT5B*** | 1.8 | wingless-type MMTV integration site family, member 5B |

**Supplementary Table 8. Genes that have significantly higher expression in the FTO obesity-risk genotypes (C/C) as compared to normal (T/T) and low in DN as compared to SC samples.** FC: fold change after white and brown differentiation, average fold change (FC) when two data considered
